# Neurophysiology of Robot-Mediated Training and Therapy: A Perspective for Future Use in Clinical Populations

**DOI:** 10.3389/fneur.2013.00184

**Published:** 2013-11-13

**Authors:** Duncan L. Turner, Ander Ramos-Murguialday, Niels Birbaumer, Ulrich Hoffmann, Andreas Luft

**Affiliations:** ^1^Neurorehabilitation Unit, University of East London, London, UK; ^2^Lewin Stroke Rehabilitation Unit, Department of Clinical Neurosciences, Cambridge University NHS Foundation Trust, Cambridge, UK; ^3^Institute of Medical Psychology and Behavioral Neurobiology, MEG Center, University of Tubingen, Tubingen, Germany; ^4^Health Division, Tecnalia Research & Innovation, San Sebastian, Spain; ^5^Ospedale San Camillo, Istituto di Ricovero e Cura a Carattere Scientifico, Venezia Lido, Italy; ^6^Clinical Neurorehabilitation, Department of Neurology, University of Zurich, Zurich, Switzerland

**Keywords:** motor cortex, spinal cord, rehabilitation, motor learning, motor adaptation

## Abstract

The recovery of functional movements following injury to the central nervous system (CNS) is multifaceted and is accompanied by processes occurring in the injured and non-injured hemispheres of the brain or above/below a spinal cord lesion. The changes in the CNS are the consequence of functional and structural processes collectively termed neuroplasticity and these may occur spontaneously and/or be induced by movement practice. The neurophysiological mechanisms underlying such brain plasticity may take different forms in different types of injury, for example stroke vs. spinal cord injury (SCI). Recovery of movement can be enhanced by intensive, repetitive, variable, and rewarding motor practice. To this end, robots that enable or facilitate repetitive movements have been developed to assist recovery and rehabilitation. Here, we suggest that some elements of robot-mediated training such as assistance and perturbation may have the potential to enhance neuroplasticity. Together the elemental components for developing integrated robot-mediated training protocols may form part of a neurorehabilitation framework alongside those methods already employed by therapists. Robots could thus open up a wider choice of options for delivering movement rehabilitation grounded on the principles underpinning neuroplasticity in the human CNS.

## Introduction

Stroke or spinal cord injury (SCI) often leaves an individual with persistent functional movement deficits that impact on independent living and quality of life, whilst putting an enormous healthcare and macro-economic burden on societies (1). Such sudden onset injury to the central nervous system (CNS) was long considered immune to treatment. However, in the last three decades a paradigm shift has occurred whereby a better understanding of recovery has highlighted the potential for re-organization of neural circuits that remain intact after stroke or SCI (2–4). Recovery involves several regions of the CNS and can spontaneously occur after stroke or incomplete SCI, that is, in the absence of specific training.

Several potential therapies may assist or guide this spontaneous recovery and include constrained induced movement therapy (CIMT), robot-mediated therapy, pharmacological treatments (e.g., selective serotonin re-uptake inhibitors), brain-machine interfaces (BMIs), goal oriented physiotherapy, epidural spinal stimulation, non-invasive cortical stimulation, electromechanical-mediated therapy, and combinations thereof (5–13). Taking two examples, both CIMT (the EXCITE trial) and robot-mediated therapy (the VA robot trial) have been demonstrated to induce better clinical outcomes than usual care following stroke (9, 13).

## Neuroplasticity in the Human Brain

Underlying many existing motor therapies is the central tenet that repetitive, progressive, and engaging practice using the affected limb induces plastic changes in neural networks subserving motor control and learning. The changes could be both functional and structural-anatomical and the neurophysiological processes by which these changes might occur, have been collectively termed as neuroplasticity (2, 3, 14, 15).

Neuroplasticity occurs at synapses and involves molecular changes in cell signaling pathways and neurotransmission; both dendritic and axonal plasticity can occur in healthy conditions and also after damage to the CNS (16–18). There are spike time-dependent changes in neuronal synaptic strength that can be demonstrated in response to high-frequency stimulation in *in vitro* and *in vivo* animal studies and which contribute to changes in neurophysiology such as increased or decreased evoked post-synaptic potentials (EPSPs) that can persist for long periods [i.e., long-term potentiation or depression; LTP and LTD (18)]. Since the pioneering studies of the 1960s and 1970s and subsequent rapid consolidation of understanding of mechanisms underpinning LTP/LTD, induced changes in synaptic strength have also been directly demonstrated *in vitro* in human tissue surgically excised from either the hippocampus or neocortical temporal lobe (19, 20). More recent studies in humans, have demonstrated analogous changes in cortical excitability following high-frequency sensory stimulation (19). Paired associative conditioning stimulation paradigms (PAS) such as non-invasive peripheral nerve stimulation paired with non-invasive transcranial magnetic stimulation (PNS and TMS respectively) as well as non-invasive weak transcranial direct current stimulation (tDCS) can also induce LTP/LTD-like changes in motor cortical excitability and are mediated by complex neurotransmitter and neuromodulatory systems in a similar manner to the original *in vitro* animal studies (21). Thus the human brain has the capacity for neuroplastic adaptation to changing environmental conditions.

The next translational step to make in favor of human neuroplasticity is to demonstrate that changes in synaptic strength resulting from these basic molecular, cellular, and neurophysiological phenomena can lead to re-organization of neural connectivity at the local small world network level, across the cerebral hemispheres, along the spinal cord segments and ultimately could occur across the whole CNS system. An approach to this is to combine neuroimaging of the whole brain (e.g., functional magnetic resonance imaging; fMRI) and site-specific non-invasive brain stimulation (e.g., tDCS on motor cortex). For example, applying unilateral anodal tDCS to motor cortex reduces resting interhemispheric cortical and contralateral intra-cortical functional connectivity (22), but increases ipsilateral motor-premotor, motor-parietal cortical functional connectivity as well as cortico-striato-thalamic functional connectivity (23, 24). Thus, the adult human CNS appears to have the capacity to adapt to artificial (e.g., tDCS) and more natural stimulation (e.g., visual or auditory stimuli), both in terms of cell-based neurophysiology and at neural network-based levels, thereby demonstrating an innate capacity to undergo neuroplasticity.

## Neuroplasticity in the Clinic

**Figure 1:**
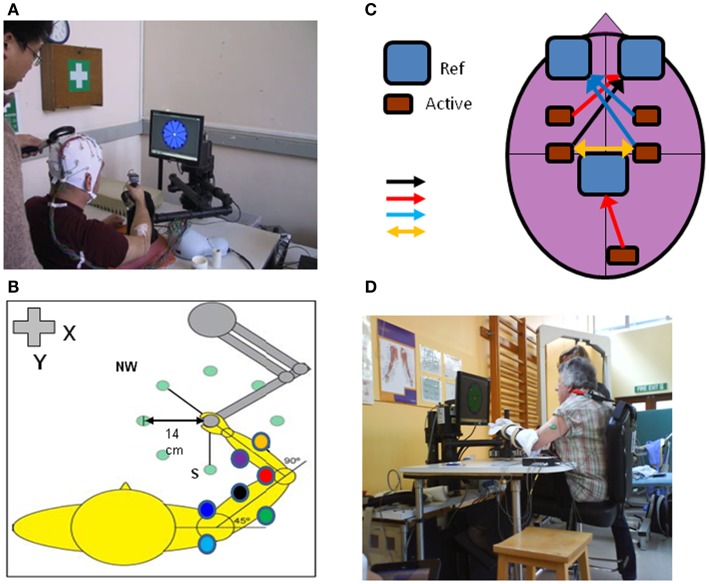
**An upper limb end-effector robotic device can be used to monitor cortical and neuromuscular responses with TMS, EEG, and EMG (electrodes placed on multiple shoulder, arm, forearm muscles) during performance of reaching movements in different directions in the *x*-*y* axis (A,B)**. The motors can be switched off to measure “free” movements or switched on to induce force fields (perturbation or resistance). Other adjunct methods of brain stimulation can be used during robot-mediated reaching movements such as tDCS **(C)**; different types of tDCS include: unilateral anodal motor cortex – black arrow, unilateral anodal premotor or visual cortex – red arrows, unilateral cathodal stimulation – blue arrows or directional stimulation – yellow arrow; Ref, reference electrode, Active, active electrode). The robotic device can be used to assist acute stroke patients in reaching motor practice in therapy or be programed to perturb motor performance to measure patient kinematic performance and muscle responses in different tasks such as position holding [**(D)**; see also Figure 4].

Several recent reviews cover general aspects of rehabilitation following stroke and SCI and the potential role of neuroplasticity in recovery processes (25–33). Here we specifically focus on the potential of robot-mediated therapy to induce neuroplasticity as evidenced by some or all of the basic phenomena highlighted. There is a growing evidence-base for neuroplasticity to occur in healthy subjects when they engage with robot devices in studies of motor learning (Figure 1).

Whether these learning mechanisms demonstrated in health also occur during rehabilitation employing robot devices for neurological recovery is not fully established in the literature, we therefore highlight some recommendations for future research rather than a meta-analysis of available evidence. We will highlight points of caution where we translate evidence for examples of robot-mediated neuroplasticity in learning in healthy adults to those individuals with CNS injury (34).

## Neuroplasticity and Robot-Mediated Learning

This perspective focuses on four elements of robot-mediated learning with respect to their potential to induce neuroplasticity in clinical populations. Evidence from studies on healthy subjects and then on neurological populations will be described and a summary on potential future research areas put forward for each element.

## Element 1: Robotic Assistance

Assistance by a robot involves the device providing a haptic interaction^1^ and there is a growing range of control strategies associated with it [(35) for a comprehensive review]. For example, this could incorporate a “haptic tunnel” for the movement path (36) in the form of forces provided by actuators to reach a movement target when the patient is not able to perform the desired range of motion (37, 38) or in the form of correct movements performed by a robot not in contact with the patient [i.e., the robot has a coaching role; (35, 39)]. Strategies correlating contingent proprioceptive and/or other sensory inputs to motor outputs also might be important for inducing neurophysiological changes (18, 40).

### Evidence for neuroplasticity: Upper limb

Recent work has demonstrated that robot-assisted wrist movements or hand grip in healthy subjects are accompanied by different frequency-dependent power changes in the electroencephalogram (EEG) in neural cortical circuits compared to voluntary wrist movement or hand grip (41, 42). Furthermore, assistive haptic feedback during a visuomotor tracking task induces region-specific changes in frequency-dependent power compared to tracking with no haptic feedback. Interestingly, there are also increases in functional connectivity (coherence) between cortical regions involved in the motor task only when assistive haptic feedback is present (43). Robot-assisted unilateral wrist movement modulates contralateral alpha and beta frequency power (desynchronization) in cortical areas that are also involved during voluntary wrist movements (41). Further, the movement-evoked potentials of voluntary and assisted (non-robotic in this case) finger movements are at similar times (35 vs. 36 ms respectively) after movement onset and are in the same current source locations (44). Substantial overlap of neural activity representation is also demonstrated for elbow flexion/extension in voluntary and torque-motor (i.e., similar to robotic) driven conditions (45). Thus (active) voluntary and (quasi-passive) robot-assisted motor tasks activate similar brain regions.

However, neuroplasticity *per se* is considered to be underpinned by progressive, challenging motor skill learning rather than merely repetitive motor tasks. Active voluntary motor skill learning with the wrist leads to more prominent increases in (i) activity in contralateral primary motor cortex, (ii) motor excitability recruitment curves, and (iii) intracortical facilitation compared to passive (torque-motor assisted) motor skill learning (46). The greater changes in motor excitability in active voluntary vs. passive motor skill learning have also been repeated for ankle flexion/extension in visuomotor tracking (47). These findings suggest that robot-assisted motor skill learning may not necessarily be as influential as voluntary motor skill learning in inducing neuroplasticity. Encouragingly however, there is evidence to suggest that re-organization of brain networks can occur after robot-assisted therapy in stroke patients both in terms of regional activation (48) and interhemispheric and intrahemispheric functional connectivity (49).

### Evidence for neuroplasticity: Lower limb

Stroke and SCI can impede the ability to walk significantly and reduce independence in living. Surprisingly therefore, there is little knowledge of the neural mechanisms underlying lower limb functional recovery; even less is known about the impact of walking rehabilitation on the neural aspects of recovery and virtually nothing is known about the impact of robot-assisted therapy of the lower limb on neuroplasticity.

Cortical control of locomotion involves a complex interplay of supraspinal circuits, spinal interneurons, and spinal reflexes. Either spinal or supraspinal (stroke) injury could bring about re-organization of all levels of the neuroaxis. A full review of the neural correlates of locomotor control in clinical populations is beyond this perspective [see Ref. (25, 50, 51)]. Nevertheless some common aspects can be demonstrated whereby unilateral hemispheric stroke effects functional (measured with TMS) and structural (measured with diffusion tensor MRI) corticospinal tract integrity and this is proportional to walking impairment (52). On the other hand, corticospinal tract integrity above an incomplete cervical spinal lesion is also reduced in terms of spinal cord area, smaller white matter volumes in pyramids and left cerebellar peduncle and smaller gray matter volume in the leg area of the motor cortex – importantly, clinical impairment was correlated with some of these functional-structural measures (53). Whilst the specific changes may be different between stroke and SCI, both types of injuries can be associated with brain re-organization.

Can these re-organizations of brain function and structure be “tuned” or enhanced by motor training? There is strikingly little information available to answer this especially in the early stages of recovery (54). Longitudinal imaging studies have documented an increase in neural activation in midbrain and cerebellum following extended aerobic walking training in chronic stroke patients [i.e., along with the post-stroke re-organization detailed earlier; (55)]. Walking velocity was correlated with midbrain and cerebellar activation, suggestive of neuroplasticity underpinning clinical improvement. Both cortical and subcortical regions appear to be involved in walking training rehabilitation intervention in chronic stroke (56, 57). The balance between cortical and subcortical neuroplasticity in these two similar study paradigms may have been due to whether proximal (55) or distal leg muscle function was tested during fMRI (56). Robot-assisted, body-weight supported walking training on a treadmill resulted in greater sensorimotor and cerebellar activation following a prolonged intervention after incomplete SCI, (58) and greater corticomotor responses to transcranial magnetic stimulation of the leg cortical representation during stroke rehabilitation (59). Finally, robot-assisted treadmill walking training (with concomitant cognitive and imagery training) increased sensorimotor neural activation and functional connectivity in a case study of adult traumatic brain injury (60).

The overall evidence that healthy motor learning and neuroplasticity is induced or facilitated by robot *assistance* is rather scarce. However, it has been suggested that robot assistance may promote motivation, because motor performance during training can be better than without assistance (34, 61). Clinically, robot-assisted therapy is effective after acute and chronic stroke (38), although not more so than intensity-matched physical therapy when a human therapist in part assists, in part motivates the patient’s own movements (9). Analysis of biomechanical aspects of motor recovery, suggest that motor learning (i.e., neuroplasticity) and not motor adaptation characterizes motor recovery after robot-assisted therapy, although this is not direct evidence gathered using neuroimaging (62). Robot-assisted therapy generally includes many thousands of repetitive movements over 1–6 months and this total intensity is required for neuroplasticity in animal models (63). Unfortunately, this intensity is rarely or never matched in other well studied therapies such as CIMT or functional electrical stimulation (FES) or indeed in usual care – the evidence for neuroplasticity following CIMT and FES is preliminary and out of the scope of this perspective [see review by (33)].

In summary, performing motor tasks with robot-mediated assistance can modulate neural activity compared to un-assisted or active voluntary movements in healthy subjects and stroke patients, although whether the patterns of modulation are similar in health and disease remains to be compared. Strong direct evidence for neuroplasticity following robot-assisted therapy is currently lacking and future work is required to identify which type of assistance is optimal for inducing neuroplasticity and thus reducing motor impairment.

## Element 2: Robotic Perturbation

Perturbing a movement, for example by applying an external force, renders it more difficult to perform. Increased difficulty adds to the intensity of training and could serve as a stronger learning stimulus; on the other hand perturbations that are too large may hinder the learning process. Several paradigms using robotic devices have been used to investigate the neuroplasticity that occurs when healthy subjects have learned to “adapt” to a perturbation during reaching or tracking movements of the hand (upper limb) or ankle (lower limb).

### Evidence for neuroplasticity: Upper limb

**Figure 2:**
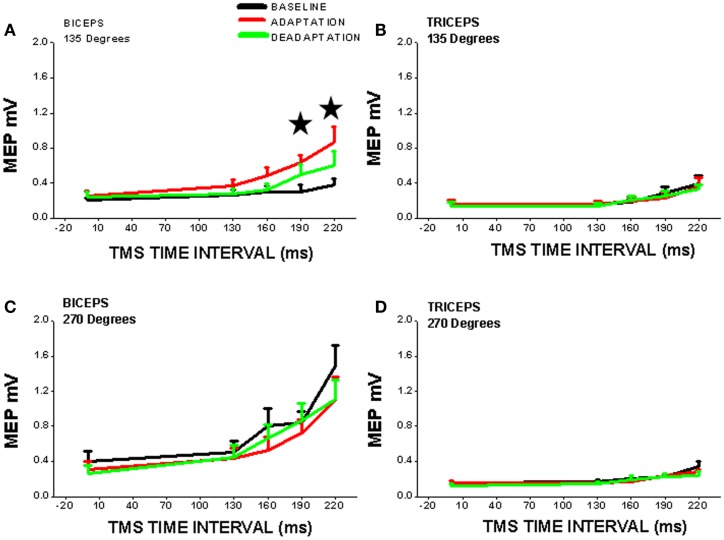
Cortical excitability of contralateral motor cortex is significantly increased (*, vs. BASELINE condition) during robot-mediated clockwise force field perturbation adaptation in healthy subjects (ADAPTATION condition cf. BASELINE and DEADAPTATION conditions). TMS was used to measure cortical excitability during the movement preparation period before reaching (TMS time interval is time after visual signal to start reach and is set at *x* = 0 on *x*-axis) to two different directions [**(A,B)** = 135°, away from the body; **(C,D)** = 270°, toward the chest] and for two different upper limb muscles [**(A,C)** = biceps; **(B,D)** = triceps]. Note that cortical excitability is only increased for one muscle (biceps) in one direction of perturbed reaching (135°), so cortical neuroplasticity is thus muscle- and direction-specific. The increase in cortical excitability precedes reaching movement and suggests that there is a change in the “internal model” of the biceps muscle within the cortex [from Ref. (65) with permission].

One common paradigm incorporates adaptation to robot-induced force fields which physically perturb ongoing arm movement (64). Changes in cortico-striatal neural activation, cortical excitability along with short interval intracortical inhibition and facilitation have been demonstrated during such adaptation processes [e.g., (65, 66); Figure 2].

Perturbation stimulates the healthy motor system to adapt, that is, to counteract the external force and can involve adaptation of predictive “feedforward anticipatory” movement or force production (an “internal model”) and adaptation of reactive “feedback” adjustments of limb movement in response to the perturbation. Adaptation is a fast process that can be distinguished from learning by repeating the same unperturbed movement over and over again (67). Whereas the latter depends on activity (use)-dependent neuroplasticity, cerebellar error-based learning mechanisms may account for adaptation of reaching during force-field perturbations (68). A shift of activation from cortico-striatal to cortico-cerebellar networks occurs while adapting to an external force field and this is associated with changes in effective connectivity amongst cortical regions in healthy humans (66, 69). Shifts in neural network activation during motor tasks persist following rest periods after force-field motor adaptation possibly indicating a “motor memory” consolidation process [i.e., neuroplasticity; (70)]. Persistent memory of motor responses is best achieved by combining error-based adaptation and use-dependent plasticity (67). Whether, this combination of healthy motor learning mechanisms is active during recovery from brain injury remains to be demonstrated. Preliminary studies however, suggest that incorporating error augmentation may be a beneficial strategy for upper limb therapy in chronic stroke patients (71, 72).

**Figure 3:**
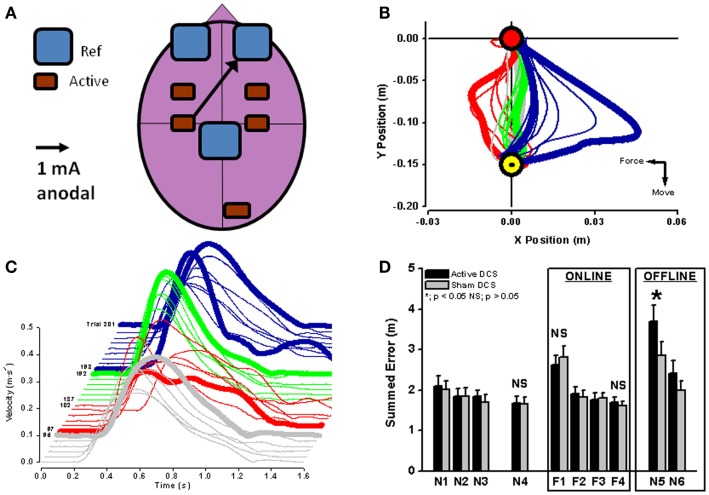
**Unilateral anodal tDCS (black arrow with the cathodal electrode applied supraorbitally; (A) was applied to contralateral motor cortex during force-field adaptation in order to augment ongoing neuroplastic changes in cortical neurophysiology (see Figure 2)**. Interestingly, *online* tDCS stimulation did not change the reduction of movement error or recovery of velocity *during* motor adaptation [red lines in **(B,C)**], but did result in a significant increase (*) in *offline* movement error once tDCS and the robot-induced force field were both switched off (blue lines in **(C,D)**; black bars in **(D)**. Reaching blocks N1–N4 and N5–N6 are without force field and reaching blocks F1–F4 are with force field perturbation. From (74) with permission).

In healthy subjects, cortical excitability of a brain region can be modulated by applying unilateral anodal tDCS during motor adaptation (Figure 3A). Cerebellar anodal tDCS during arm-reaching adaptation to visuomotor rotation results in a faster rate of adaptation (73), whereas retention of the “offline motor memory” of adapted behavior is enhanced by anodal stimulation of the primary motor cortex [visuomotor rotation – (73); robot-induced force field – (74); Figures 3B–D].

Recent work using *in vitro* motor cortex brain slices has suggested that tDCS interacts with coincident low frequency stimulation (in possible analogy to afferent activity accompanying movement during human motor adaptation) to increase BDNF secretion and TrkB activation (75). Both of these induced molecular changes are stimulation(activity)-dependent and characteristic of synaptic neuroplasticity (18). In order to augment neuroplasticity during robot perturbation training in clinical populations in this way, future effort is required to determine the optimal selection of the stimulated brain region (cerebellar vs. cerebral), site of tDCS related to location of injury (ipsilesional vs. contralesional), type of tDCS (anodal vs. cathodal), and the type of robot-mediated therapy (unilateral vs. bilateral). Indeed, studies using single hemisphere tDCS and robot-mediated *bilateral assistive* therapy in stroke patients did not demonstrate clinically significant effects on motor recovery (76). Repetitive TMS is a similar technique to tDCS in the sense of modulating cortical excitability, however currently it has not been used either with robot-mediated motor adaptation in healthy subjects or with robot-assisted therapy in clinical populations.

### Evidence for neuroplasticity: Lower limb

The concept of perturbation has also been applied to the lower limb in gait training. Short-term motor adaptation can occur in healthy subjects during walking when one limb operates in a force-field environment and the behavioral adaptation is associated with changes in cortical excitability (77). Indeed, when tDCS is applied to cerebellum in healthy subjects, the rate of motor adaptation to split-belt treadmill walking can be increased or decreased depending on the modality of tDCS [i.e., anodal vs. cathodal respectively; (78)].

In clinical studies involving patients with cerebellar degeneration, predictive feedforward components of motor adaptation were impaired, whereas reactive feedback components were not impaired, when the patients were walking on a split-belt treadmill with the two belts – one for each leg – running at different velocities (79). On the other hand, stroke patients with cerebral damage could adapt in a similar fashion to healthy control subjects, when performing the same split-belt walking paradigm (80). These differential findings in clinical populations suggest that the cerebellum may be more important than the cerebral cortex in perturbation adaptation in the lower limb. Lasting improvement remains to be demonstrated in large clinical studies but the first trials suggest that gait asymmetry in chronic stroke can be ameliorated by split-belt walking training (81). However the long-term neuroplastic changes underlying the adapted behavior in both healthy subjects and clinical groups are unknown, but deserve future investigation.

In summary, perturbation-based robot-mediated therapy following neurological injury has not received the attention that assistive robot-mediated therapy has and there is a lack of direct comparative evidence to suggest one is better than the other currently. Further, while there is some evidence to suggest that modulation of motor *and* sensory neural circuits occurs (82) during motor adaptation in health and disease, caution should be kept in mind when translating evidence from learning/adaptation in healthy subjects to stroke compared to cerebellar degeneration, for example. Nevertheless, future studies using combinations of assistive and perturbation-based motor adaptation (71) with or without adjunct non-invasive brain stimulation may be worthwhile clinically and elucidate the impact of robot-mediated perturbation on neuroplasticity *per se*.

## Element 3: Adding Virtual Reality to Robot-Mediated Therapy

Virtual reality (VR) has been combined with a robotic training device in gait training after stroke and can significantly augment gait improvements more than robot therapy alone (83). The impact of VR on robot-induced gait improvements after stroke is manifest as increases in force and power via improvement of ankle motor control (84). If used appropriately, VR can represent to the stroke patient certain bio-signals related to gait performance such as heart rate or force/torques at lower limb joints and thus stimulate conscious control of precision movement (85).

Unfortunately a significant number of stroke survivors often see little progress in their training because improvement is slow and post-stroke depression may devalue reward. Several rewarding features can be built into training robots, such as those providing immediate feedback about movement errors as well as delayed rewards, for example by collecting points or virtual money. VR can provide an excellent framework for reward presentation. Reward regions in the brain have been proposed to contribute to motor skill learning in an animal model via dopaminergic pathways (86). Recent studies in healthy humans have suggested that primary motor cortex, which participates in motor learning, also responds to rewarding of successful behavior – increased reward was correlated with greater paired-pulse inhibition using TMS (87). Furthermore, motor skill learning when performed in positive-reward conditions led to a prolonged long-term retention of a motor memory, whereas neutral or punishment-related skill learning did not (88). Hence, any strategy that enhances reward signals for correct movement sequences via VR or other additional technologies during robot-mediated therapy may have a clinical benefit. In summary, adding VR to robot-mediated therapy remains to be explored both in terms of neuroplasticity and clinical application. Additionally, the type of visual stimuli used in VR-robot environments requires further investigation.

## Element 4: Interfacing the Brain with a Robotic Device

The idea of using a combination of a BMI and robots for rehabilitation has been explored in several recent studies and is justified by the absence of rehabilitation therapies for paralyzed and severely impaired stroke patients. These patients cannot benefit from existing therapy since residual movement ability is generally necessary. Rehabilitation robotic devices hold the potential to bridge the gap between the intention to move (i.e., in the CNS) and actual movement of an orthosis or robot device without the need of a limb (89–91). In this section, we deal with neurological patients only, as the fundamental development of BMI systems is outside the perspective scope. However, feasibility studies on healthy subjects are often required initially to investigate synthetic and neurophysiological artifacts when linking the brain of the BMI-user and movements of a limb or robot (40).

Stroke patients can acquire control over a hand orthosis (opening/closing of the hand) by volitionally modulating sensorimotor frequency-dependent rhythms in the lesioned hemisphere. While most of the patients were able to learn controlling the orthosis via the BMI, clinical scales used to measure hand function showed no improvement after training (92). In another study, two groups of sub-acute stroke patients who received either standardized robotic training or BMI-driven robot training demonstrated the ability to improve performance by using motor imagery in the ipsilesional motor cortex (93). A single case study reported recovery of a severely affected chronic stroke patient using a combination of BMI-robot therapy and physiotherapy (94). Functional and anatomical neural correlates of functional clinical outcome measures of recovery were evaluated in a multimodal imaging approach, whereby increased lateralization of neural activity occurred in the ipsilesional hemisphere and white matter re-organization occurred in the ipsilesional corticospinal tract (94). The effectiveness of brain-robot interfaces in stroke rehabilitation may be improved by “closing the feedback loop,” whereby haptic feedback enables ipsilesional sensorimotor loops to be re-activated (95). Interestingly, the contralesional hemisphere can also be activated during attempted reaching tasks in severely affected chronic stroke patients (96). This suggests that several brain regions have the capability to interact with a robot-effector and the injured brain region can be “bypassed” (97). This would be a good example of how robot based therapeutic design can be built on an understanding of principles of neuroplasticity and the functional connectivity between several brain regions during recovery from stroke. For example, a recent study demonstrated that the motor imagery component of brain-robot interface training can augment changes in functional connectivity in chronic stroke patients beyond that induced solely by robot-assisted therapy (98). Lastly, a very recent controlled study proved the efficacy of a rehabilitation paradigm using BMI and behavioral physiotherapy in chronic stroke patients, closing the loop between brain signals related to movement intention and that same movement via a BMI controlled robotic orthosis (99). In this double-blind feasibility study, 32 chronic stroke severely paralyzed patients (without residual finger extension) received 18 training sessions. One group received contingent BMI-training: ipsilesional cortical-desynchronization was linked to movements of a robotic orthosis fixed to the paralyzed limb. The control group (sham) received the same training, but the movements of the orthotic device were randomized and independent of cortical desynchronization. Both groups received identical behavioral physiotherapy after every BMI-session. The experimental group showed a significant improvement in Fugl-Meyer upper limb motor scores, BMI control, increased muscle activity, and control in the paralyzed hand and arm and lateralization of brain activation toward the ipsilesional hemisphere when compared to the control group.

In summary, this rapidly expanding field is yielding significant methodological steps forward in the design of upper and lower limb rehabilitation using BMI with robots or other hybrid approaches such as acquired self-control of brain activity (12, 50, 100, 101). The neurophysiological mechanisms, measured for example with TMS, underpinning motor improvement; the role of neuroplasticity, and the clinical value of these combined therapy approaches remain to be fully explored.

## Critical Summary of the Potential for Robot-Induced Neuroplasticity in Neurological Recovery from Injury

**Figure 4:**
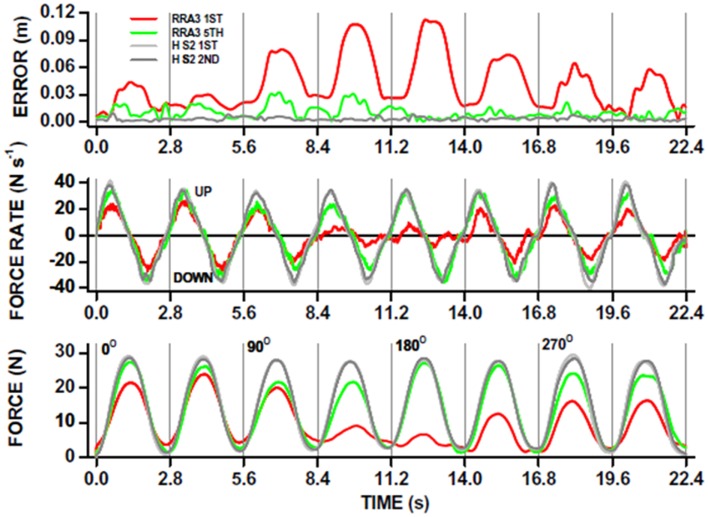
**Robot-mediated perturbations can be used to evaluate acute stroke patient motor performance in a “holding” task**. The patient is instructed to hold the joystick in the middle of a computer screen and the robot exerts “pulling” forces to the joystick (see also Figure 1D). This acute stroke patient undertook 20, 1 h therapy sessions, each including ∼1000 robot-assisted reaches to peripheral targets on a computer screen in different directions. The ability to hold the joystick in a central position whilst the robot applied “pulling” forces in different directions, was measured before (red traces; RRA3 first) and after (green traces; RRA3 fifth) the robot-assisted therapy program. The overall *x*-*y* position error (top panel) was significantly reduced after robot-assisted therapy toward that measured in healthy subjects (gray traces; HS2 first and second). Note that position holding performance was direction-specific in this patient. The kinematic improvement in position holding was the result of increases in kinetic force production (bottom panel) and also the rate of force production (UP) and relaxation (DOWN; middle panel) toward that of healthy subjects. From Ref. (117) with permission.

The use of robot-mediated therapy for augmenting recovery from neurological injury is now becoming more attractive as evidence for cost effectiveness of robot-mediated therapy is becoming stronger (9, 102). As robot device use increases, it is likely that an increasing range of therapy strategies will be designed [see Ref. (35) for examples]. The incorporation of neuroimaging and monitoring of neurophysiology alongside robot-mediated therapy is in its infancy in comparison (one example of kinematic analysis is shown in Figure 4). Nevertheless, there is some suggestion that changes in neural activation and functional connectivity can be associated with robot-mediated movement and therapy.

## Paradoxes to Resolve in Robot-Induced Neuroplasticity

However, critically assessing the evidence for neuroplasticity induced by robot use has highlighted several large gaps in our knowledge and some paradoxes that should be addressed (Table 1).

**Table 1:** **Levels of evidence for neuroplasticity in robot-assisted therapy and robot motor learning employing various elements**.

	Robot-assisted therapy	Robot motor learning
Assistance	Low	Low
Perturbation	Low	High
Reward	Low	Low
Brain-machine interface	Low	Low

The first paradox is that whilst robot-*assisted* therapy is the most frequently robot-based intervention for neurological injury, we know little about the neural correlates of this type of movement (41, 42) and even less on whether an assistance control strategy induces neuroplasticity *per se* (42, 43, 48, 49). It could be argued that assisting movements (i.e., similar to passively moving the limb) might require less neural activation than actively engaging with a voluntary effort to move a limb, even when it is not possible to move it. Certainly, passive movement elicits a lower neural activation “intensity” and altered regional pattern compared to active movement or motor imagery of the same type of task in stroke patients; note this was not the case in healthy subjects performing the same task and thus translating healthy to neurological concepts warrants caution (103).

The second paradox is that whilst we know a significant amount about the neural correlates of motor learning and neuroplasticity induced by robot-mediated perturbations in healthy subjects, this type of robot usage is rarely used in neurological therapy (71). The neuroplasticity in response to adaptation to robot perturbations of movement can be substantial and widespread. Importantly, recently this type of neuroplasticity induced by motor adaptation was demonstrated to be long lasting even at the single neuron level (104). Future work is required to assess whether robot perturbation type therapy induces neuroplastic changes which correlate with clinical outcome.

The third paradox is that whilst cortico-striatal neural activation is modulated during and after robot perturbing motor adaptation in healthy subjects (66) and reward circuits may be involved in motor skill learning (86, 88), the impact of motivation/reward on neuroplasticity during robot-based motor adaptation is not known in either healthy subjects or in neurological patients. This might be important to consider, because there is a growing development of autonomous control of robot-related therapy characteristics (e.g., level of force used; type of assistance and so on) as home-based robot therapy becomes more probable (105–109). Increasingly, the human-robot interaction will require bi-directional input in terms of the patient being able to achieve goals/rewards to maintain high adherence to therapy whilst using the robot on the one hand and the robot being driven optimally by patient performance on the other.

The fourth paradox involves the interaction of brain and robot without the need for an actuating human limb. Using brain signals to drive a robot device directly to undertake everyday tasks and to induce motor rehabilitation is feasible following severe stroke (99, 110, 111). The changes that occur in neuronal cell tuning properties and firing co-variance, spike timing across neural networks, and spectral changes during the period of learning how to drive the robot by thought or movement intention alone, suggest that neuroplasticity occurs (112). However, it could be that there is a substantial change in neural output over a prolonged time “practicing” the specific task such that the same neuron groups become resistant to learning other new tasks (or their tuned responses that drive the robot task in the first instance suffer from interference). Learning different types of robot-mediated motor perturbations certainly demonstrate patterns of interference which degrade performance and rather rapidly so (113, 114); thus BMI induced re-organization might inhibit/interfere with learning future new tasks if the re-organization becomes too “entrenched” in neural circuits involved in movement. Long-term studies of BMI use to study possible neuroplastic changes are required to answer these questions.

## Conclusion

There are several large gaps in our knowledge on the neural correlates of effective robot-mediated therapy (Table 1). The rapid advances in robot design, but more importantly neuroimaging techniques compatible with robotics (e.g., Figure 1) will catalyze the next steps in understanding the role of robot-mediated neuroplasticity in successful recovery from brain injury.

Robots are more than aids for or simply replacements of therapists to deliver movement therapy. From a technical perspective, robots can be excellent research tools, because they provide ways to standardize rehabilitative training, to precisely monitor recovery of motor function in patients [Figure 4; (62, 115–117)] and to control protocols for subjective human influence. The concepts described in this perspective suggest future work for developing training methods grounded in neurophysiological principles that can be delivered by robotic devices to optimally stimulate neuroplastic processes and learning in the CNS. Most likely no single concept will be the single solution for all patients. Rather combinations will provide a highly individualized training that is delivered in a repetitive and standardized fashion, for example tDCS and robot-mediated therapy (63). This will produce robot-based assessment measures that are comparable across patients with different motor disorders or at different time points of their lifespan (e.g., childhood vs. adult; (118)) in the clinic and at home (119).

## Conflict of Interest Statement

The authors declare that the research was conducted in the absence of any commercial or financial relationships that could be construed as a potential conflict of interest.

## Footnotes

^1^Relating to the sense of touch, in particular relating to the perception and manipulation of objects using the senses of touch and proprioception: *haptic feedback devices create the illusion of substance and force within the virtual world*. Oxford Dictionaries, Oxford University Press, Oxford, England 2012.
